# Automatic recognition of adrenal incidentalomas using a two-stage cascade network: a multicenter study

**DOI:** 10.1080/07853890.2025.2540596

**Published:** 2025-08-07

**Authors:** Xiao Xie, Sheng-Xiao Ma, Xiang-De Luo, De-Ying Liao, Dong Han, Zhi-Peng Huang, Zhi-Hua Chen, Xian-Ping Li, Bo Li, Shi-Di Hu, Yan-Jun Chen, Peng-Fei Liu, De-Zhong Zheng, Hui Xia, Cun-Dong Liu, Shan-Chao Zhao, Ming-Kun Chen

**Affiliations:** ^a^ Department of Urology, Third Affiliated Hospital, Southern Medical University, Guangzhou, China; ^b^ The Third Clinical College, Southern Medical University, Guangzhou, China; ^c^ School of Mechanical and Electrical Engineering, University of Electronic Science and Technology of China, Chengdu, China; ^d^ Shanghai Artificial Intelligence Laboratory, Shanghai, China; ^e^ Clinical Data Statistics Center, Third Affiliated Hospital, Southern Medical University, Guangzhou, China; ^f^ Department of Urology, Yuancheng People’s Hospital of Guangdong Province, Heyuan, China; ^g^ Department of Urology, Longmen People’s Hospital of Guangdong Province, Huizhou, China; ^h^ Department of Endocrinology, Third Affifiliated Hospital, Southern Medical University, Guangzhou, China; ^i^ Department of Radiology, Third Affifiliated Hospital, Southern Medical University, Guangzhou, China; ^j^ Department of Clinical Laboratory, Third Affifiliated Hospital, Southern Medical University, Guangzhou, China; ^k^ Department of Cardiology, Third Affifiliated Hospital, Southern Medical University, Guangzhou, China

**Keywords:** Adrenal incidentalomas, deep learning, machine learning, radiomics, CT

## Abstract

**Background:**

The incidence of adrenal incidentalomas (AIs) is increasing yearly. The early discovery of AIs is helpful to better manage adrenal diseases, especially subclinical primary aldosteronism, Cushing’s syndrome and pheochromocytoma.

**Methods:**

In this multicenter retrospective study, a total of 778 patients from three different medical centers were assessed. The two-stage cascade network consisted of a 3D Res-Unet network for adrenal gland segmentation and a classifier for determining the presence of AIs. The segmentation network was mainly evaluated by the Dice similarity coefficient (DSC), and the classifier was evaluated by the area under the receiver operator characteristic curve (AUC), accuracy, sensitivity, and specificity. The Delong test was used to compare the classification performance between the cascade network and manual segmentation.

**Results:**

A total of 443 patients were randomly assigned in a 7:3 ratio, stratified sampling, to train and valid sets of the model development cohort, and 335 patients from the three centers were included in the test cohort. In the validation set, the AUC of the model for identifying left AI was 88.15%, and the AUC of the model for identifying right AI was 87.90%. There was no significant difference between model performance and manual segmentation of AIs (*p* > 0.05). In the test cohort, the cascade network achieved AUC of more than 80% and accuracy of more than 75% for both left and right adrenal glands.

**Conclusions:**

The two-stage cascade network based on a deep learning algorithm can be used for automatic recognition of AIs in nonenhanced CT from different centers.

## Introduction

Adrenal incidentalomas (AIs) are defined as adrenal masses that are fortuitously discovered during imaging examinations for nonadrenal ailments [1]. About 20%–50% of these AIs to secrete the hormone cortisol, lead to metabolic disorders, impaired immune function, and cerebrovascular disorders, anxiety and depression, and even ultimately lead to increased mortality [2–4]. Therefore, the early, timely and accurate detection of AIs holds tremendous significance in enhancing patients’ quality of life and extending their lifespan. With the widespread clinical application of CT imaging technology, the detection rate of AIs has experienced substantial advancements in recent years, rising from approximately 0.6% in 1982 to 2.3% in 2019 [5–7]. However, the detection rate of AIs in autopsy series reports is close to 9.0% [8]. The discrepancy may be due to the fact that AIs are similar to other incidental tumors, such as pituitary and thyroid tumors, which frequently go unnoticed due to their diminutive size and insufficient clinical attention [9,10]. Consequently, devising effective strategies for identifying AIs has emerged as a pressing concern.

Radiomics, a quantitative technique that uses features from medical images, has found widespread application in disease diagnosis, clinical staging, treatment evaluation and prognosis assessment. Its efficacy has been substantiated by numerous studies [11–13]. However, the implementation of radiomics heavily relies on human effort to delineate images, which consumes many human resources. Moreover, the delineation process is often influenced by physician experience and fatigue, thereby introducing an element of subjectivity [14]. Therefore, the automatic segmentation adrenal area become one of the key steps in automatically recognize the AIs. Compared to other organs, the segmentation of the adrenal gland poses unique challenges due to its bilateral asymmetry, small size, indistinct boundaries, and close proximity to surrounding tissues. Previous studies have explored various segmentation algorithms for the adrenal gland, primarily employing computer vision methods in limited datasets [15–17]. However, in data-rich and heterogeneous clinical data, algorithms that target specific data and adjust parameters may be difficult to apply in clinical scenarios [18]. Deep learning algorithms have gained popularity in the medical field due to their exceptional learning and generalization capabilities. Recent studies have reported a DSC of approximately 72.98% for multiorgan segmentation models applied to adrenal gland segmentation using extensive datasets [19]. However, the current model segmentation performance of adrenal gland is far weaker than other organs, limiting its clinical application [19]. Hence, optimizing deep learning algorithms to stably segment adrenal glands and combining radiomics to establish machine learning models for AIs screening can effectively save labor costs and avoid the influence of subjectivity.

The objective of this study is to develop a cascade network based on deep learning technology for automatic recognition of adrenal incidentalomas on unenhanced CT scans. Furthermore, the performance of the network will be tested using multicenter data.

## Materials and methods

### Collection of patient data

**Figure 1.**
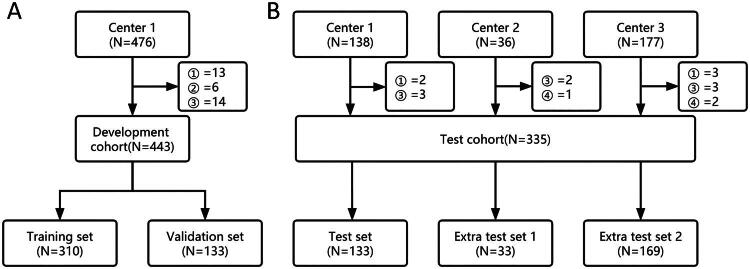
Development cohort (A) and test cohort (B) filter flowchart. Exclusion reason: ① Repeated CT scan. ② After surgery in the adrenal region. ③ Poor CT image quality (including position changes or imaging artifacts). ④ CT image format mismatch.

To ensure patient privacy protection, rigorous data desensitization measures were implemented for all cases included in this study. The model development cohort originated from the Imaging Data Center of the Third Affiliated Hospital of Southern Medical University (Center 1). A retrospective collection of 476 adrenal nonenhanced CT images from January 2018 to August 2021 was conducted. This study included the time period for the complete adrenal CT scan images, images in the report for a complete description of the adrenal glands. Repeated CT examination images, images after adrenal region surgery, poor CT quality, and CT image format non-DCM format were excluded. Following the inclusion and exclusion criteria (Figure 1A), a total of 443 nonenhanced CT images belonging to 443 patients were ultimately obtained (20,446 slices). Imaging reports and clinical information for the patients were collected. The image data were uniformly converted to Nifty format, and subsequently, the adrenal area was manually delineated by three junior urologists using ITK-SNAP [20] 3.8.0, following standardization procedures. The delineation results were thoroughly examined by senior radiologists. During the delineation process, clinical information and imaging reports were not accessible. Due to the use of structured adrenal reporting in Center 1, original imaging reports were used as the basis for reporting AIs for all data in the development cohort. Because data from Centres 2 and 3 were not reported through structured adrenal reporting, the data were independently reviewed by a senior radiologist and urologist. If consensus was not reached, a third senior urologist was invited to intervene to obtain the final results. According to the current guidelines for the management of AIs, further examination and treatment are not recommended for AIs less than 1 cm. Therefore, the shortest adrenal diameter greater than 1 cm is defined as the cut-off value [21]. The development cohort was divided into a training set and a validation set, utilizing a 7:3 stratified random sampling method based on the presence of Als within the entire image. A total of 351 patients were assessed in the test cohort from three centers, including the test set from the 2017 Imaging Center database of Center 1, extra test set 1 from the Longmen People’s Hospital of Guangdong Province (Center 2), and extra test set 2 from the Yuancheng People’s Hospital of Guangdong Province (Center 3). Following the application of inclusion and exclusion criteria (Figure 1B), a total of 335 nonenhanced CT images were obtained (17068 slices).

### Deep learning segmentation model

ln this study, the segmentation model utilized Medical Open Network for AI (MONAI) version 0.8 and PyTorch version 1.8.0 as the underlying framework. All programming tasks were executed on a 12 GB NVIDIA GTX3060 GPU, and the code can be accessed on GitHub (https://github.com/XieXiao1997/AIsRecognizedTCN). We first used a 3D segmentation network to extract the adrenal glands from high-resolution CT images, where we extended the classical 3D U-Net in several aspects. First, since CT images inherently possess three-dimensional characteristics and each cross-section holds a high degree of information interaction, a 3D U-Net network containing residual units, known as the 3D residual U-Net network, with lower sampling layers of 32, 64, 128, 256 and 512, was utilized in this study [22,23]. Second, this network employed a 3D convolutional layer instead of the conventional 2D convolutional layer. Moreover, a residual unit was incorporated into the updown sampling process of the traditional U-Net network to deepen the network structure and mitigate overfitting. Third, considering that the foreground-background imbalance resulting from the small size of adrenal volumes could drop the network performance, a combination loss function of the Dice loss [24] and focal loss [25] was employed to boost the prediction quality. In addition, to reduce the impact of the different patients, centers and imaging protocols on the final performance, we employed some general preprocessing strategies for the nonenhanced adrenal CT images. First, we reformatted all CT images into a standard direction PLI (posterior, left, inferior). Then, we resampled all images into a fixed spacing of [1.0, 1.0, 3.0], which is the median spacing of the development cohort. After that, we truncated the CT intensity values with the abdominal window (W:350 Hu, L:40 Hu) and rescaled them to the range of 0 to 1. During the training stage, data augmentation techniques were employed to mitigate overfitting, which included the random cropping of a patch from preprocessed images and the addition of random Gaussian noise and random rotation angles to the patch. Then, the 3D segmentation network was trained with the following details: The input patch size was [96, 96, 96]; an initial learning rate of 3e-4 was set to facilitate early convergence; a multistep learning rate annealing strategy was adopted to prevent network oscillation, wherein the learning rate decayed to 0.9 times the previous rate every 20 epochs; and the Adam optimizer was employed for training, utilizing 200 epochs. Images were postprocessed and verified through inference at the end of each training cycle. In this study, considering the variations in the number of cross-sectional data across different centers, the images were partitioned into four subvolumes with dimensions [96, 96, 96]. These subvolumes were carefully selected to encompass both the adrenal region and background information, enabling the utilization of the sliding window paradigm during both the training and inference stages. To address potential issues such as noise or edge discrepancies that may arise from the direct output of the segmentation network, postprocessing of the images was conducted during the inference stage. Specifically, the connected domain of each region was calculated to eliminate noise, and any fragmented regions were filled with appropriate hole-filling techniques. Furthermore, during each training epoch, model parameters demonstrating a lower loss function compared to the previous epoch were saved and retained.

### Classification model of AIs

**Figure 2.**
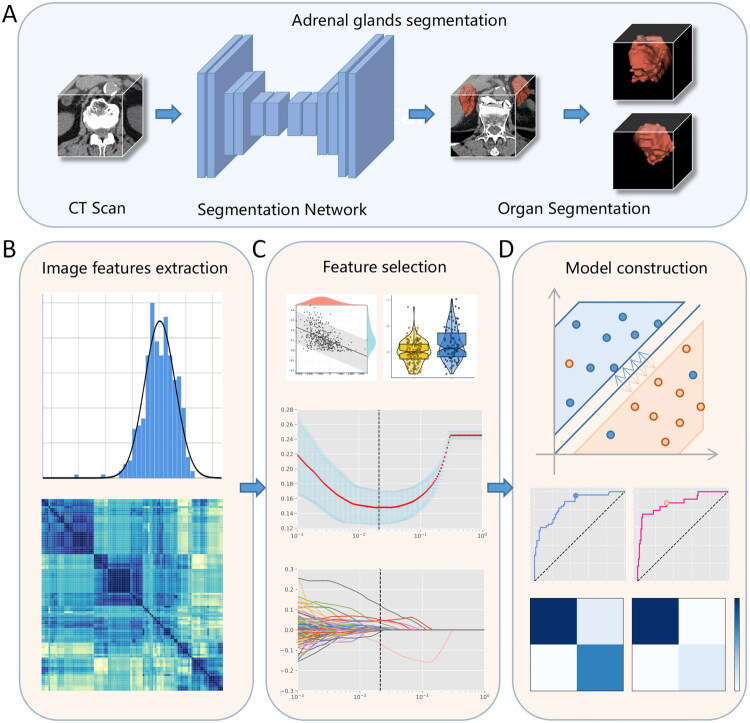
The flow chart of the cascade network for recognition of adrenal incidental tumors was constructed. After preprocessing, the adrenal glands were segmented using CT scan without contrast images and inference through 3D Res-Unet network (a). Then, bilateral adrenal features (B) were extracted respectively, and feature engineering was carried out to screen out key features of adrenal incidentalomas (C), and finally transferred to two classification models to identify adrenal incidentalomas(D).

In this study, the classification model utilized Scikit-Learn [26] 1.0.1 as the underlying framework. A pipeline was devised to transfer the segmented adrenal image data to the AI classification model, extract image features from the left and right adrenal regions, and subsequently filter the extracted features. The reduced-dimensional characteristics were then inputted into the classifier to determine the presence or absence of AIs within the adrenal glands. To acquire more suitable classifiers, a comprehensive evaluation of various commonly used machine learning models was conducted to determine the optimal classifier for AI classification. The detailed data flow diagram is depicted in Figure 2, and the corresponding code is accessible on GitHub (/Classfication). In addition, we have provided four sample datasets to assess the performance of our developed two-stage cascade network (/Sample).

### Model performance evaluation

The segmentation model was evaluated by comparing the automatically segmented adrenal glands with manual annotations using the validation set. Several metrics were employed to quantify the performance of the segmentation model, including the DSC (Dice similarity coefficient) [27], IOU (intersection over union) [28], RVE (relative volume error) [29] and HD (Hausdorff distance) [30]. The DSC and IOU metrics primarily assess the internal filling of the segmented region, with higher values indicating better performance. RVE evaluates the volume error between the segmented region and the true region, with smaller values representing better performance. HD, on the other hand, is more sensitive to the boundary of the segmented region, and smaller values represent better performance. To mitigate the impact of small outliers on the calculation of the HD index, the evaluation index was modified by employing the 95th percentile HD quantile (i.e. HD95) instead of HD itself. The classification model was evaluated using both the validation set and the complete test cohort. The performance of the classification model was assessed using the receiver operating characteristic curve (ROC) and the corresponding area under the ROC curve (AUC) [31]. Moreover, the accuracy, sensitivity, and specificity of the model were calculated by determining the optimal cut-off value that maximizes the Youden index [32].

### Statistical analysis and visualization

Statistical analysis in this study was performed using Scipy 1.7.1 [33] and SPSS 25.0 (SPSS Company in Chicago, Illinois, USA). The mean and standard deviation of the segmentation model evaluation index were reported. Evaluation metrics of the classification model were reported as rates, accompanied by 95% confidence intervals when applicable. Differences between continuous groups were assessed using the Mann–Whitney test, while the chi-square test was used for categorical groups. Student’s t test was employed to compare the differences between the evaluation indexes of the segmentation model. The DeLong test was utilized to compare the AUCs of two different classification models [34]. A two-tailed *p* value < 0.05 was considered statistically significant. Result visualization was performed using matplotlib 3.4.3 and 3D Slicer [35] 5.0.3.

## Results

### Patient cohort and characteristics

This study included 778 patients (Table 1). In the developmental cohort, the median age of 443 patients was 47 (IQR 37–56) years, and 182 (41.08%) were women. Among these patients, 220 (49.66%) had left or right AIs. There were 143 patients (32.28%) with left AI only, 30 patients (6.77%) with right AI only, and 47 patients (10.61%) with bilateral AIs. The model development cohort was randomly divided into the training set (*N* = 330) and the validation set (*N* = 133), and there were no significant differences in sex, age or location of AIs between the two sets of patients (*p* > 0.05). The test cohort consisted of 335 patients, and statistical tests could not be performed due to the absence of demographic information after data desensitization at Center 2. There were 204 patients (60.90%) with left or right AI in the test cohort, including 145 patients (43.28%) with left AI only, 22 patients (6.57%) with right AI only, and 37 patients (11.04%) with bilateral AIs. Notably, the distribution of data varied among the three centers in the test cohort, with extra test set 2 including more patients with AIs and a higher prevalence of left-sided AIs.

**Table 1.** Demographic characteristics and adrenal gland characteristics in the model development and test cohort.

Characteristics	Development cohort				Test cohort				
	Training set	Validation set	*p*-value#	All patients	Test set	Extra test set 1	Extra test set 2	*p*-value*	All patients
Number	310	133	–	443	133	33	169	–	335
Gender			0.55					–	
Female	124(40.00%)	58(43.61%)		182(41.08%)	54(40.60%)	–	63(37.28%)		117(34.93%)
Male	186(60.00%)	75(56.39%)		261(58.92%)	79(59.40%)	–	106(62.72%)		185(55.22%)
Unknown	–	–		–	–	33(100.00%)	–		33(9.85%)
Age, Median [IQR], years	46[37, 55]	48[36, 58]	0.27	47[37, 56]	49[37, 64]	–	52[43, 63]	–	50[36, 62]
Gland characteristics			0.85					<0.01	
Normal	156(50.32%)	67(50.38%)		223(50.34%)	65(48.87%)	15(45.45%)	51(30.18%)		131(39.10%)
Only with left AI	101(32.58%)	42(31.58%)		143(32.28%)	46(34.59%)	11(33.33%)	88(52.07%)		145(43.28%)
Only with right AI	19(6.13%)	11(8.27%)		30(6.77%)	5(3.76%)	2(6.06%)	15(8.88%)		22(6.57%)
Bilateral AI	34(10.97%)	13(9.77%)		47(10.61%)	17(12.78%)	5(15.15%)	15(8.88%)		37(11.04%)
Examination characteristics $			1.00					<0.01	
Normal	156(50.32%)	67(50.38%)		223(50.34%)	65(48.87%)	15(45.45%)	51(30.18%)		131(39.10%)
With AI	154(49.68%)	66(49.62%)		220(49.66%)	68(51.13%)	18(54.55%)	118(69.82%)		204(60.90%)

Note: Between training set and validation set. * Between the three test set. $ On either side.

### Performance of the adrenal segmentation network

**Figure 3.**
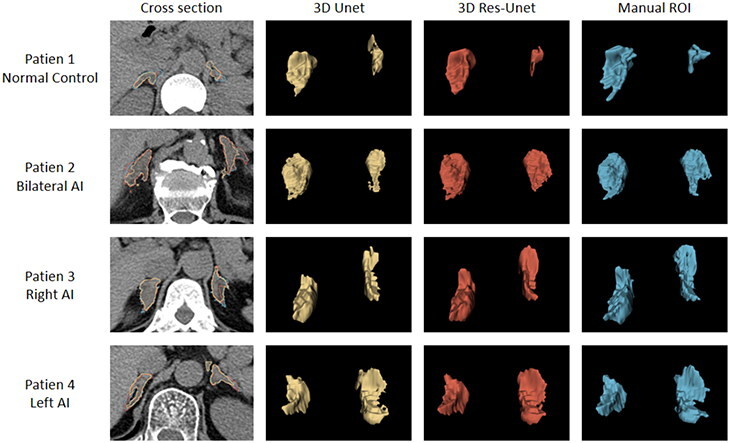
Visual comparison of network segmentation performance between 3D Res Unet and 3D-Unet. For cross-sectional visualization in the first column, yellow, red, and blue borders represent 3D Unet, 3D Res-Unet, and manual segmentation, respectively.

After training, the performance of the 3D Res-Unet network and 3D Unet network on the validation set was compared (Table 2). In terms of the whole image, the 3D Res-Unet network achieved a higher DSC and IOU of 85.94 ± 5.74% and 75.75 ± 8.19%, respectively, while the 3D Unet network achieved lower values of 82.42 ± 8.14% and 70.82 ± 10.53%, respectively. Regarding the average relative volume error and the Hausdorff distance between point sets, the 3D Res-Unet network showed better performance with values of 7.20 ± 8.75% and 3.17 ± 3.13 mm, respectively, compared to the 3D Unet network with values of 12.93 ± 13.06% and 4.59 ± 5.14 mm, respectively. For the unilateral adrenal gland, the segmentation performance of the 3D Unet network was slightly weaker on the left side (81.05 ± 12.49%) than on the right side (83.58 ± 8.6%). However, the 3D Res-Unet network demonstrated similar segmentation capability for both sides (left: 85.48 ± 8.46%, right: 86.41 ± 5.87%). Overall, the 3D Res-Unet network outperformed the 3D Unet network in terms of both whole image segmentation and unilateral gland segmentation (*p* < 0.05). In addition, the adrenal gland segmentation of multiple patients in the validation set was visualized using both models (Figure 3). The 3D UNet network exhibited poor segmentation performance in certain regions, misclassifying nonadrenal tissues as adrenal tissues in all four patients. Examples include omitting the lateral branch of the left adrenal gland (Patient 2), missegmentation of the left adrenal gland (Patient 3), and misidentification of tissues such as blood vessels and diaphragms as adrenal tissue (Patients 1 and 4). In contrast, the 3D Res-Unet network had fewer misjudgment cases and provided more refined processing of adrenal gland details, resulting in segmentation closer to manual segmentation of the adrenal gland.

**Table 2.** Comparison of segmentation performance in validation set between 3D Res-Unet and 3D Unet network.

Evaluation **#**	DSC (%)	IOU (%)	RVE (%)	HD95 (mm)
3D Unet Network				
Whole Image	82.42 ± 8.14	70.82 ± 10.53	12.93 ± 13.06	4.59 ± 5.14
Left adrenal gland	81.05 ± 12.49	69.59 ± 14.23	16.26 ± 17.70	4.98 ± 5.56
Right adrenal gland	83.58 ± 8.63	72.57 ± 10.70	12.30 ± 12.91	3.12 ± 2.88
3D Res-Unet Network				
Whole Image	85.94 ± 5.74	75.75 ± 8.19	7.20 ± 8.75	3.17 ± 3.13
Left adrenal gland	85.48 ± 8.46	75.42 ± 10.79	9.18 ± 11.16	3.44 ± 3.53
Right adrenal gland	86.41 ± 5.87	76.49 ± 8.31	8.53 ± 13.15	2.48 ± 2.33

Abbreviations DSC = Dice similarity coefficient, IOU = Intersection over union, RVE = Relative volume error, HD = Hausdorff distance.

### Performance of the Cascade network in identifying AIs

**Figure 4.**
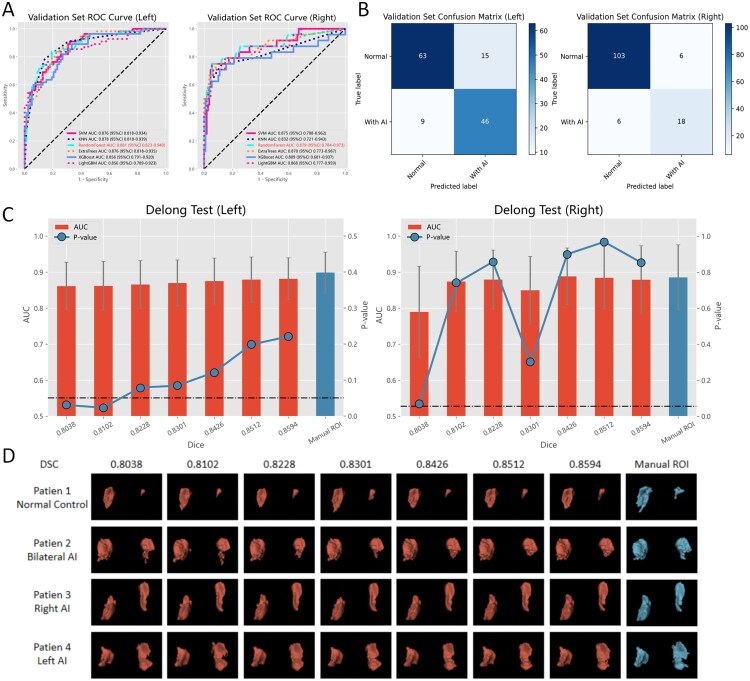
(A)The ROC curves of multiple machine learning models identifying AI in the validation set, with the best performing model shown in red font. (B) Confusion matrix for the classification model with the best performance at the cut-off threshold. (C) The bar and error bar represent AUC and 95% CI, respectively. The line shows the Delong test *p*-value compared to the manual ROI. (D)Image segmentation visualization results under different DSC, red represents automatic ROI, blue represents manual ROI.

Following feature screening, a total of 24 key imaging features for left AI classification and 6 for right AI classification were identified. The performance of various machine learning models was compared using the validation set (Figure 4A), and the random forest model exhibited the highest AUC for both left AI classification and right AI classification (left: 88.15%, 95% CI 82.34%–93.98%, right: 87.90%, 95% CI 78.49%–97.31%). Consequently, the random forest model was selected as the classifier for AIs. The optimal partitioning thresholds were determined using dynamic partitioning (left 0.38, right 0.52). Under these thresholds, the left AI classification model successfully classified 109 out of 133 patients (81.95%) with a sensitivity of 83.64% and specificity of 80.77%. Similarly, the right AI classification model achieved a correct classification rate of 121 out of 133 patients (90.98%), with a sensitivity of 75.00% and specificity of 94.50% (Figure 4B). In addition, a comparison was made between the performance of AIs classification models using manual segmentation and automatic segmentation (best segmentation performance, Table 3). The AUC values for manual segmentation were 89.91% (95% CI 84.29%–95.52%) for left AI classification and 88.55% (95% CI 79.52%–97.58%) for right AI classification. There was no statistically significant difference in the performance of AIs classification models between manual and automatic segmented adrenal glands (*p* > 0.05). To explore the relationship between the performance of the automatic segmentation model and the AIs classification model, different segmentation efficiency models were compared with manual segmentation as a control (Figure 4C). When the DSC exceeded 82.28% for left AI classification and 81.02% for right AI classification, no statistically significant difference was observed between the automatic segmentation model and the manual adrenal segmentation for AIs classification. This indicates that the constructed method maintained satisfactory accuracy and stability. In addition, visualization of adrenal gland segmentation was performed for different DSC cases (Figure 4D).

**Table 3.** The recognition performance of AI based on manual ROI and automatic segmentation.

Methods	Acc (%)	AUC (%)	AUC 95% CI	Sens (%)	Spec (%)	Threshold
Manual ROI						
Left	84.21	89.91	84.29–95.52	78.18	89.74	0.46
Right	91.73	88.55	79.52–97.58	70.83	98.17	0.66
Auto segmentation						
Left	81.95	88.15	82.34–93.98	83.64	80.77	0.38
Right	90.98	87.90	78.49–97.31	75.00	94.50	0.52

### Multicenter validation of the AIs recognition Cascade network

**Figure 5.**
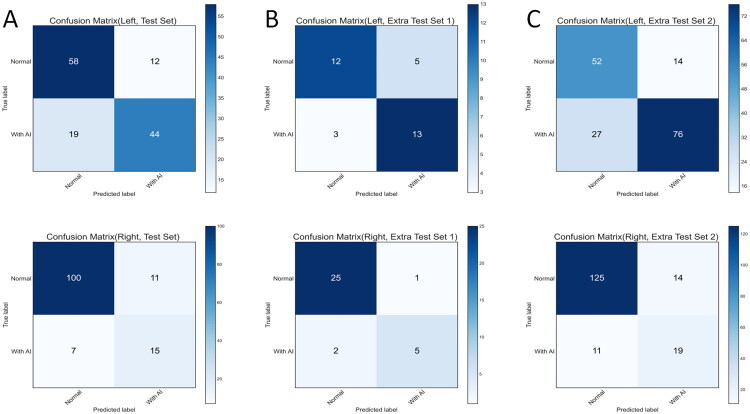
Confusion matrix for adrenal incidentalomas identification in the test cohort. (A)–(C) represent test set, external test set 1, and external test set 2, respectively.

Upon completion of training, the cascade network of AIs recognition was validated using the test cohort from three different centers. In the model validation cohort, the best cut-off value obtained from the model development cohort was used for truncation, and the confusion matrix of each dataset was output (Figure 5). The sensitivity, specificity and other indicators were calculated by a confusion matrix (Table 4). In the test set from the same center as the model development cohort, the AUC for identifying left AI reached 84.15% (95% CI 77.22%–91.08%). Out of 133 left adrenal glands, 102 were correctly classified (76.67%). The sensitivity and specificity were 69.84% and 82.86%, respectively. For right AI, the AUC was 81.04% (95% CI 68.78%–93.30%), and 115 out of 133 right adrenal glands were correctly classified (86.47%). The sensitivity and specificity were 68.18% and 90.09%, respectively (Figure 5A). In extra test set 1, which had a small sample size, the AUC identifying left AI was 84.74% (95% CI 71.83%–97.65%). Out of 33 left adrenal glands, 25 were correctly classified (75.76%), with a sensitivity of 81.25% and specificity of 70.59%. The AUC identifying right AI was 91.21% (95% CI 78.27%–100.00%), with 30 out of 33 right adrenal glands correctly classified (90.91%). The sensitivity and specificity were 71.43% and 96.15%, respectively (Figure 5B). In extra test set 2, which had a larger sample size, the AUC for identifying left AI was 80.68% (95% CI 74.11%–87.25%). Out of 169 left adrenal glands, 128 were correctly classified (75.74%), with a sensitivity of 73.79% and specificity of 78.79%. The AUC for identifying right AI was 81.87% (95% CI 72.66%–91.09%), and 144 of 169 right adrenal glands were correctly classified (85.21%). The sensitivity and specificity were 63.33% and 89.93%, respectively (Figure 5C). In summary, the developed model achieved an AUC of over 80% and an accuracy of 75% in the task of classifying AI in both the left and right adrenal glands within the test cohort from three different centers.

**Table 4.** Performance of the cascade model in the recognition of AI in test cohort.

Dataset	Test set		Extra test set 1		Extra test set 2	
	Left	Right	Left	Right	Left	Right
Acc (%)	76.69	86.47	75.76	90.91	75.74	85.21
AUC (%)	84.15	81.04	84.74	91.21	80.68	81.87
AUC 95% CI	77.22–91.08	68.78–93.30	71.83–97.65	78.27–100.00	74.11–87.25	72.66–91.09
Sens (%)	69.84	68.18	81.25	71.43	73.79	63.33
Spec (%)	82.86	90.09	70.59	96.15	78.79	89.93

## Discussion

At present, the prevalence of AIs is steadily increasing, posing a significant public health concern. In response to this issue, our study aimed to address the automatic recognition of AIs in non-contrast CT scans by developing a two-stage cascade network based on deep learning algorithms. The cascade model comprised two distinct components. The 3D Res-Unet model was employed to achieve precise segmentation of the adrenal gland. Subsequently, a machine learning classifier was devised to effectively differentiate between the presence and absence of AIs. To test the stability of the cascade network we developed, the performance of the cascade network was evaluated using data obtained from three different medical centers.

In our study, we employed the 3D Res Unet network for adrenal gland segmentation, achieving a DSC of 85.94% in a validation set of 133 cases. Notably, the traditional 3D UNet network achieved only 82.42%. The significant improvement demonstrated by the 3D Res-Unet network highlights its superiority over the 3D Unet network. These results indicate that our segmentation network achieves good performance for adrenal gland segmentation tasks.

Numerous studies have reported AIs classification models based on segmentation algorithms. Early studies utilized various machine learning algorithms for AIs screening [36,37]. While these studies achieved promising detection results, they often faced challenges related to limited sample sizes. In the latest study by Cory et al. a model for AIs detection based on venous phase images of contrast-enhanced CT scans was presented. This model achieved 69% sensitivity and 91% specificity in a secondary validation set comprising 1,902 adrenal glands, including 75 AIs [38]. However, it should be noted that the model developed in this study primarily relied on the venous phase of the enhanced CT scan. From an imaging perspective, the adrenal gland appears clearer in the venous phase compared to the nonenhanced CT, which may result in better segmentation performance. However, there are certain limitations associated with this approach. In clinical practice, during the initial diagnosis, nonenhanced adrenal CT scans are typically performed, and contrast-enhanced CT scans are not commonly used as a screening scan [10]. Additionally, contrast-enhanced scans can significantly increase patient costs, and some patients may be unable to undergo enhanced scanning due to allergies to contrast media. Therefore, in the clinical scenario of AIs recognition, a development model based on nonenhanced CT scans seems to be more appropriate.

**Figure 6.**
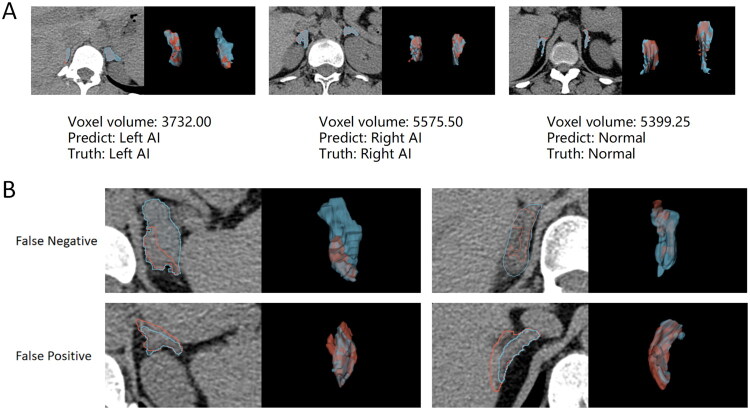
(A) Case of adrenal gland segmentation with smaller voxels. (B) False positives/false negatives in complex cases. The left side of each case is a cross-sectional view, and the right side is a 3D visualization. Red represents the automatically segmented adrenal gland and blue represents the manually segmented adrenal gland.

Based on our experience, in the cascade system, the segmentation model’s performance can directly impact the classification model. Therefore, we examined the relationship between segmentation performance and classification performance. When selecting the optimal segmentation model (DSC = 85.94%), the left and right AUCs of the AI classifier in the validation set were 88.15% and 87.90%, respectively. However, when selecting a model with decreased segmentation performance (DSC = 80.38%), the left and right AUCs of the AI classifier dropped to only 86.14% and 78.98%, respectively, indicating a decrease in classification efficiency. By further refining the DSC level, we observed that when the DSC was above 82.28%, the classifier’s performance did not show a statistically significant difference from manual labelling. For this reason, preserving a portion of the segmentation performance reserve was crucial for maintaining the stability of the classification model. Nine of the top 10 adrenal voxels with the smallest sum of bilateral adrenal voxel volumes in the valid data (ranging from 3732.00 to 6329.25 voxels) were well segmented and classified. The segmentation and classification of three representative small adrenal glands were shown in Figure 6A. Nevertheless, in the analysis of misclassification cases, we found that even with the retention of segmentation performance reserve in this study, the segmentation effect of certain complex cases remained flawed, leading to false positive/false negative results (Figure 6B).

In the medical field, the application of clinical models must also consider their generalization ability, even though it is challenging to quantify. Generalization ability refers to the model’s capacity to achieve stable prediction performance in different scenarios [39]. To explore the generalization capability of our developed model, we trained the model using data from one center and validated it using datasets from three different centers. Our cascade model, developed for the recognition of AIs, consistently demonstrated an AUC of over 80% and an accuracy of over 75% in each of the three validation datasets, regardless of the left or right adrenal glands. This suggests that our model possesses robust automated recognition capabilities with the ability to generalize well across data from different centers. The integration of our AI model into clinical workflows has the potential to significantly improve patient care. By automatically flagging potential AIs on routine CT scans, our model could increase detection rates, particularly for smaller lesions that might be overlooked. This could lead to earlier intervention for functional tumors and more timely follow-up for indeterminate masses. Furthermore, by reducing the workload on radiologists, the model could allow for more focused attention on complex cases, potentially improving overall diagnostic accuracy.

However, there were limitations to our study. First, it was a retrospective study and lacked prospective validation. Integrating the network into a central imaging system could be considered to further explore the clinical utility of the network. Second, although we tested the cascade network in three centers, the sample size of our study was still relatively small, and the number of participating centers was limited. Future efforts should focus on increasing the sample size and testing the model in more centers to enhance the generalizability of our findings. In addition, in the development cohort, adrenal glands were delineated by three different primary urologists, which may have introduced interobserver differences and biases. It was challenging to assess the impact of these differences on the results. Despite preserving a portion of the segmentation capacity, some patients still experienced poor segmentation performance, such as the relatively close adhesion to the surrounding tissues shown in Figure 6B (especially the right adrenal gland), which consequently affected the classification model results. Therefore, it is necessary to further adjust the segmentation model and improve the segmentation accuracy. Finally, the size threshold for the inclusion of AIs within our study was 1 cm, a criterion that has been frequently employed in previous investigations. However, it has been suggested in some studies that adrenal tumors smaller than 1 cm may still exhibit endocrine function (sporadic nodular adrenocortical disease) [40], thereby posing a challenge in terms of effective patient screening.

In conclusion, our study introduced a two-stage cascade network for the automatic recognition of AIs on nonenhanced CT scans. By eliminating the need for manual delineation, our network offers advantages such as human resource savings and reduced subjectivity in image interpretation. However, further efforts are necessary to enhance adrenal segmentation efficiency and expand the sample size for more robust and generalizable results.

## Data Availability

Due to ethical review and data confidentiality restrictions, data from this study cannot be shared except for the sample data provided in the github web. The sample data may not be used in other studies without a written request and permission from the corresponding author.

## Data availability statement

The program code built in Python in this study is available on github (https://github.com/XieXiao1997/AIsRecognizedTCN).

## Abbreviations

Adrenal incidentalomas: AIs
Dice similarity coefficient: DSC
Area under the receiver operator: AUC
Medical Open Network for AI: MONAI
Intersection over union: IOU
Relative volume error: RVE
Hausdorff distance: HD
Confidence interval: CI
